# Changes in Quality of Cold-Pressed Rapeseed Oil with Sinapic Acid Ester-Gelatin Films during Storage

**DOI:** 10.3390/foods11213341

**Published:** 2022-10-24

**Authors:** Dobrochna Rabiej-Kozioł, Alicja Tymczewska, Aleksandra Szydłowska-Czerniak

**Affiliations:** Department of Analytical Chemistry and Applied Spectroscopy, Faculty of Chemistry, Nicolaus Copernicus University in Toruń, Gagarina 7, 87-100 Toruń, Poland

**Keywords:** active packaging, gelatin films, sinapic acid esters, cold-pressed rapeseed oil, antioxidant activity, shelf life

## Abstract

In recent years, cold-pressed rapeseed oil (CPRO) has become an attractive health-beneficial product and a promising alternative to olive oil. However, a high level of unsaturated fatty acids renders a CPRO more susceptible to oxidative deterioration. Therefore, the effect of new gelatin strips with polyvinyl alcohol (GEL-PVA) and sinapic acid esters (ethyl sinapate—ESA; octyl sinapate—OSA, and cetyl sinapate—CSA) on the oxidative stability, antioxidant activity (AA), and total phenolic content (TPC) in CPRO samples was analyzed during accelerated storage. In addition, the antioxidant properties of the GEL-PVA films loaded with sinapic acid esters were studied. Interestingly, the CPRO stored in an amber glass marasca bottle containing GEL-PVA-ESA strips for 14 days had the highest AA, while the antioxidant potential was the lowest for oil without film strips. Furthermore, oil samples stored in bottles containing GEL-PVA-ESA and GEL-PVA-OSA strips for 14 days had higher antioxidant properties than the AA and TPC in fresh CPRO. Moreover, synchronous fluorescence (SF) spectroscopy and excitation-emission matrix (EEM) fluorescence spectroscopy were applied for the evaluation of changes in the main fluorescent components in CPRO samples during the accelerated storage. Overall, this study revealed that GEL-PVA films incorporated with sinapic acid esters enhanced the antioxidant potential of CPRO and delayed oxidative degradation by releasing amphiphilic antioxidants into the oil.

## 1. Introduction

Today, consumers are choosing less-processed, healthy foods. Therefore, cold-pressed oils have begun answering the public demand for less processed foods due to the lack of chemicals, heat treatments, and refining processes (degumming, neutralization, bleaching, and deodorization) used compared to refined oils [1]. Awareness of the cold pressing process, which involves a mechanical, solvent-free oil extraction from oilseeds, may be desired and reflected in the consumer acceptability of such a final product. Despite the beliefs and social expectations regarding cold pressing, these oils, in some respects, contain fewer health-promoting ingredients than “processed” oils.

Rapeseed oil is the most commonly used oil in food-producing, cooking, frying, and direct consumption in Poland. Moreover, due to its valuable ingredients, rapeseed oil is readily used all over the world as an alternative to health-promoting olive oil [2,3]. Rapeseed oil consists of triacylglycerols (TAG = 94.4–99.1%) and non-TAG fractions. However, other specific compounds, such as phospholipids, tocopherols, phenolic compounds (mainly sinapic acid and its derivatives), carotenoids, chlorophylls, sterols (mainly brassicasterol, campesterol, stigmasterol, β-sitosterol, and avenasterol), free fatty acids, and mono- (MAG) and diacylglycerols (DAG), present in the non-TAG fraction of oil could have an important role in these beneficial effects. The TAG primarily is composed of monounsaturated fatty acids (oleic acid, C18:1 *n*-9 = 61%), polyunsaturated fatty acids (linoleic acid, C18:2 *n*-6 = 21% and α-linolenic acid, C18:3 *n*-3 = 11%), and small amounts (about 7%) of saturated fatty acids. Additionally, the ratio (2:1) of linoleic acid (C18:2 *n*-6) to linolenic acid (C18:3 *n*-3) is desirable in the human diet. However, the high level of polyunsaturated fatty acids in rapeseed oil increases its susceptibility to oxidation [1].

Recent studies have demonstrated that cold-pressed oils have lower oxidative stability than solvent-extracted oils, hot-pressed oils, or oils pressed from microwave pretreated rapeseed [1,4,5,6]. Cold-pressed rapeseed oil (CPRO) is more susceptible to autoxidation than refined rapeseed oil due to the higher amounts of hydroperoxides and free fatty acids in unrefined oil [4]. Additionally, the chlorophylls present in CPRO prevent the oil from autoxidation and the formation of free radicals in the dark, but they are prooxidants in photosensitized oxidation [7]. Moreover, CPRO is also known for its significant nutritional attributes due to its naturally occurring antioxidants such as polyphenols (mainly sinapic acid derivatives), tocopherols, and carotenoids. Hydrophilic and hydrophobic sinapic acid derivatives, namely, free sinapic acid, sinapine (the choline ester of sinapic acid), and canolol (a decarboxylated product of sinapic acid), are the major phenolics found in crude rapeseed oil in amounts of about 16 μg/g, 19 μg/g, and 244 μg/g, respectively [8]. Sinapic acid constitutes over 73% of free phenolic acids and has various biological properties, including antioxidative potential, anxiolytic-like effects, anti-inflammatory properties, and antibacterial activity. Moreover, it is a potent inhibitor of peroxynitrite and thus can be used to protect cellular defense activity against diseases involving peroxynitrite [9].

Unfortunately, the amounts of these natural hydrophilic and hydrophobic antioxidants are insufficient to protect the oil from oxidation. Researchers have explored several strategies for overcoming the weak oxidative stability of cold-pressed oils. One of them is the addition of synthetic or natural antioxidants [5]. Much of the interest in hydrophilic and hydrophobic antioxidants present in plant extracts and bioactive peptides from animal sources for the stabilization of edible oils was developed because of the trend to minimize or avoid the use of synthetic antioxidants having adverse health effects on humans [10]. On the other hand, many environmental and technological factors such as temperature, presence of oxygen, light, moisture, and concentrations of anti- and prooxidant components affect the antioxidant activity and cause various chemical changes, oxidation, and loss of antioxidants in vegetable oils. In addition, many natural antioxidants have reduced efficacy compared to their synthetic counterparts. This poor performance has been attributed to the hydrophilic nature of phenolic acids and low solubility in the lipid substrate, in addition to other factors. Thus, grafting a lipophilic moiety to these hydrophilic compounds could be a good alternative to enhance their solubility and antioxidant properties in lipidic media [11]. The lipophilization of sinapic acid using alcohols with increasing alkyl chain lengths enhanced its hydrophobicity and resulted in multifunctional amphiphilic molecules with a modified hydrophilic-lipophilic balance (HLB) and lipophilicity [12,13,14]. The HLB value is a numerical correlation that provides the antioxidant, solubilizing, and emulsifying properties of these new molecules, namely phenolipids, between oil-water and water-oil phases [15]. Phenolipids with a low HLB and high logarithm partition coefficient (log P) value are antioxidants that are more soluble in oil, while those with a high HLB and low log P value have a stronger affinity to water. According to the McGowan scale, the HLB value of these amphiphilic molecules depends upon the lengths and structures of the alkyl groups. The HLB values significantly decrease in a linear relationship when the number of carbon atoms in the straight alkyl group increases. Moreover, linear relationships were observed between the log P and the HLB values calculated by the procedure given by McGowan [16]. For this reason, we found an increase in log P values of sinapic acid esters in the order of 2.26 < 5.34 < 8.87 for ethyl sinapate (ESA), octyl sinapate (OSA), and cetyl sinapate (CSA), respectively [14]. In contrast, these phenolipids were characterized by relatively low HLB values (8.06, 5.21, and 1.41 for ESA, OSA, and CSA, respectively); hence, they are more soluble in oil.

On the other hand, the components with different HLB values affected the stability of film-forming emulsions, microstructure, physical, mechanical, antioxidant, and antimicrobial properties of active films as well as the release of essential oils from the composite films [17,18,19,20].

It is known that the amphiphilic nature of antioxidants affects the chemistry of lipid oxidation due to their appropriate localization in the multiphase system. The minor amphiphilic components (MAG, DAG phospholipids, sterols, and free fatty acids) naturally occurring in CPRO or produced as oxidation products (hydroperoxides, aldehydes, and ketones) can entrap water droplets. Recent research assumed that the heterogeneous lipid-water systems affected the start of lipid peroxidation due to a larger lipid surface area, increased O_2_ diffusion, and a possibility of solubility in water oxidation initiators [21]. Therefore, the HLB values of amphiphilic antioxidants are important factors to consider when selecting antioxidants to be applied in highly unsaturated lipid systems.

Recently, innovative concepts such as active antioxidant packaging, encapsulation, or nanotechnology (nanoparticles and nanoemulsions) have been proposed to fortify oils with antioxidants. These approaches allowed the sustained release of antioxidants during the storage of oil-based products [22,23]. Moreover, the proposed strategies for releasing exogenous antioxidants into oil avoid limitations related to the direct incorporation of antioxidants by better protecting them from environmental factors and improving the solubility and stability of these active molecules [24]. However, the addition of raw propolis powder with high antioxidant properties to packaging demonstrated a heterogenous appearance and unsatisfactory physical properties [22]. Therefore, further studies are needed in order to clarify the functional differences between the direct addition of antioxidants to oil and the use of active packaging or edible coatings rich in antioxidants.

Gelatin (GEL), as a mixture of peptides and proteins, is commonly used in food packaging because of its good film-forming ability, emulsifying and gas barrier properties, high strength, transparency, availability, and relatively low-cost biodegradability, biocompatibility, and non-toxicity. Unfortunately, it suffers from weak mechanical resistance, has a poor water vapor barrier, and is not water-resistant. However, the modifications of gelatin films by incorporating functional materials and active ingredients such as polyvinyl alcohol (PVA), antioxidant compounds, and plant extracts to reduce the limitations of gelatin film application have been reported by other authors [23,24,25,26,27,28].

On the other hand, polyphenols have often been used as antioxidant agents in active packaging films to inhibit food oxidation. Nevertheless, polyphenols have a common phenolic feature but also structural diversity which causes their antioxidant properties to differ. Although hydroxycinnamic acid derivatives were mixed with a biopolymer matrix for the preparation of active materials for food packaging, to the best of our knowledge, there has been no reference on studying the effect of the addition of sinapic acid alkyl esters to GEL-PVA films in the context of CPRO storage. Three hydroxycinnamic acid derivatives, ESA, OSA, and CSA, were selected as antioxidant molecules to be added to GEL-PVA films due to their different HLB properties. Therefore, it seems worth considering the application of the active sinapic acid ester-gelatin sealing films to enhance the antioxidant properties of CPRO samples and inhibit their oxidative degradation during accelerated storage. Therefore, the purpose of the present work was to estimate the antioxidant properties of GEL-PVA films incorporated with sinapic acid alkyl esters and the antioxidant activity (AA) of the oils by two modified analytical methods, 2,2-diphenyl-1-picrylhydrazyl (DPPH) and cupric reducing antioxidant capacity (CUPRAC). Moreover, the total phenolic content (TPC) and oxidative status of the CPRO samples were evaluated and discussed. Additionally, synchronous fluorescence (SF) spectroscopy and excitation-emission matrix (EEM) fluorescence spectroscopy were used to observe the most characteristic qualitative changes in the stored CPRO samples.

## 2. Materials and Methods

### 2.1. Chemicals and Oil Samples

All chemicals used in the study were of analytical or HPLC grade and procured from Merck Life Science (Poznań, Poland). Redistilled water was used for the preparation of solutions.

Eleven bottles of CPRO in the original packaging (amber glass marasca bottle, 250 mL) were kindly provided by a local vegetable oil factory and stored at 4 °C in the dark until the analysis.

### 2.2. Preparation of Active Films

Synthesis procedures of sinapic acid alkyl esters (ESA, OSA, and CSA) were described in our previous works [14,29].

**Figure 1:**
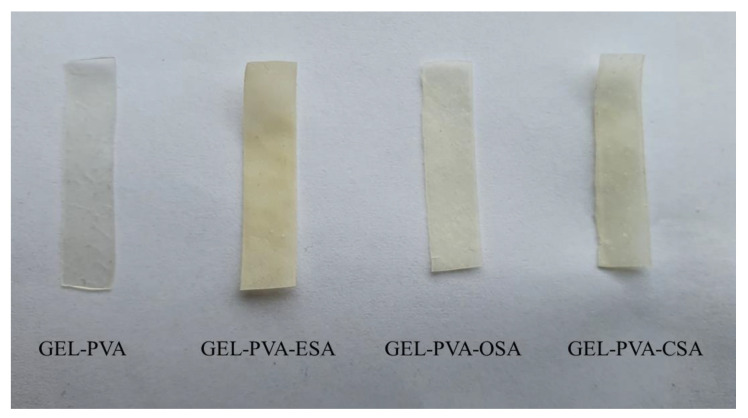
The visual appearance of the control gelatin-polyvinyl alcohol (GEL-PVA) film without ester and GEL-PVA films containing different sinapic acid esters (GEL-PVA-ESA—ethyl sinapate, GEL-PVA-OSA—octyl sinapate, and GEL-PVA-CSA—cetyl sinapate).

The concentrations and ratios of GEL, PVA, and glycerol for the preparation of active films were established based on our previous study [26]. The GEL solution (5% *w/v*) was prepared by dissolving GEL in redistilled water at 70 °C for 20 min. Then, PVA (5% *w/v*) was dissolved in redistilled water at 80 °C for 2 h. Before being added to film-forming solutions (FFS), each sinapic acid alkyl ester (1 g) was dissolved in 10 mL of ethanol. The final FFS was obtained by mixing 50 mL of GEL solution, 30 mL of PVA solution, 3 mL of glycerol, 10 mL of each sinapic acid alkyl ester (ESA, OSA, and CSA), and 7 mL of redistilled water under magnetic stirring at 60 °C for 20 min and sonicated for 3 min. The four FFS named GEL-PVA, i.e., the control without ester, GEL-PVA-ESA, GEL-PVA-OSA, and GEL-PVA-CSA, were poured (35 mL) into Petri dishes (9 cm in diameter) and left to dry at room temperature for 48 h. After drying, the obtained films (Figure 1) were peeled off from the casting surface.

### 2.3. Thickness of Prepared Films

The thickness of the films was measured by an electronic digital thickness gauge (Insize, Suzhou, China). Measurements were done with a precision of 0.001 mm at ten randomly selected parts of the films.

### 2.4. Antioxidant Properties of Active Films

The prepared films were cut into smaller pieces, weighed (0.02−0.03 g), and placed in glass tubes. After the addition of 10 mL of methanol, samples were agitated using a laboratory shaker SHKA 2508-1CE (Labo Plus, Warszawa, Poland) for 30 min. Then, extracts were collected by decantation. Antioxidant properties of the obtained film extracts were determined using the DPPH and CUPRAC methods previously described in detail [25,26].

#### 2.4.1. DPPH Method

In brief, 0.1 mL of each film extract was dissolved in 1.9 mL of methanol. Next, 0.5 mL of DPPH methanolic solution (304.0 μmol/L) was added and the obtained mixtures were shaken vigorously. These solutions were incubated in the darkness at ambient temperature for 15 min and the absorbance was measured at 517 nm against a reagent blank (2 mL of methanol + 0.5 mL of DPPH methanolic solution) using a UV-Vis spectrophotometer U-2900 (Hitachi High-Technologies Corporation, Tokyo, Japan).

The calibration curve, %DPPH = (558.15 ± 4.55)$c_{\mathrm{TE}}$ + (0.38 ± 0.30), was prepared using working solutions of 6-hydroxy-2,5,7,8-tetramethylchromane-2-carboxylic acid (Trolox—TE) in methanol between 0.02 and 0.10 µmol/mL with a determination coefficient, R^2^ = 0.9995.

#### 2.4.2. CUPRAC Method

In this procedure, 0.3 mL of each film extract, 2 mL of 0.01 mol Cu(II)/L, 2 mL of neocuproine solution (0.0075 mol/L), 2 mL of ammonium acetate buffer (pH = 7), and 3 mL of ethanol were transferred into 10 mL volumetric flasks and made up to volume with redistilled water. The obtained solutions were kept at 22 °C for 30 min. The absorbance was measured at 450 nm against a reagent blank (2 mL of CuCl_2_, 2 mL of neocuproine solution, 2 mL of ammonium acetate buffer, and 3 mL of ethanol, made up to 10 mL with redistilled water).

The calibration curve, with line equation $A_{450}$ = (11.27 ± 0.14)$c_{\mathrm{TE}}$ − (0.0097 ± 0.0057) and R^2^ = 0.9992, was constructed using a TE solution in methanol as standards in concentrations that ranged from 0.006 and 0.07 μmol/mL.

### 2.5. Accelerated Storage of Cold-Pressed Rapeseed Oil in Modified Packaging

The four prepared films, GEL-PVA, GEL-PVA-ESA, GEL-PVA-OSA, and GEL-PVA-CSA, were cut using a razor blade into strips 70 mm to 10 mm in dimension. Four strips of each film were immersed in CPRO and packed in the original amber glass marasca bottles.

**Figure 2:**
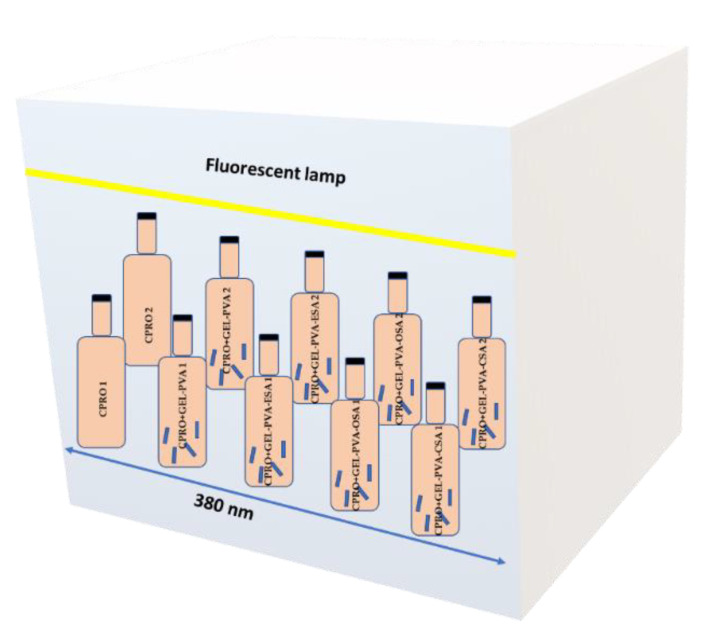
Position of bottles containing cold-pressed rapeseed oil (CPRO) without and with film strips in an incubator with the location of a fluorescent lamp.

The accelerated shelf-life experiment was conducted according to the procedure described in our previous work [30]. Briefly, two bottles with original packaged CPRO (CPRO1 and CPRO2) and eight bottles containing the four film strips prepared from the four different polymer films (the same film strips were immersed into two original bottles) were placed in an incubator (Elkon CWE-2a, Łódź, Poland) at 40 °C and under light (fluorescent lamp, T5 8W F8W/33 GE, power of luminous flux = 385 lm, length 380 nm) installed at a distance of 300 mm from the incubator shelf. The distance between the bottles was 25 mm. The position of bottles inside the incubator is presented in Figure 2. The experiment lasted 2 weeks, and the samples were drawn once a week. These conditions allow simulating real storage of CPRO in retail places or households for the recommended 6-month shelf-life period.

### 2.6. Analysis of the Oxidation Status of Cold-Pressed Rapeseed Oil

The primary and secondary oxidation products expressed as peroxide value (PV) and anisidine value (p-AnV) were analyzed by the official methods ISO 3960:2017 [31] and ISO 6885:2016 [32], respectively. The degree of oil hydrolysis was assessed by measuring the acid value (AV) using the ISO 660:2020 method [33]. Extinction coefficients (K_232_ and K_268_) were determined according to the ISO 3656:2011 method [34]. The absorbances of the oil samples dissolved in n-hexane (1% solutions) were measured at 232 and 268 nm in a 1-cm cell path length against pure n-hexane using a UV-Vis spectrophotometer U-2900 (Hitachi High-Technologies Corporation, Tokyo, Japan).

### 2.7. Analysis of the Antioxidant Properties of Cold-Pressed Rapeseed Oil

Before measuring the antioxidant properties of the CPRO samples, methanolic extracts of oils were prepared according to the previous procedure [30]. In brief, 2.00 g of each oil was extracted with 5 mL of methanol, and extraction was carried out in an SHKA 2508-1CE shaker (Labo Plus, Warszawa, Poland) for 30 min. Then, the samples were stored at –20 °C for 30 min to separate the extracts from the oils. Extractions were repeated three times, and the combined extracts were transferred quantitatively into glass bottles.

The AA and TPC in the obtained oil extracts were determined spectrophotometrically using three modified methods: DPPH, CUPRAC, and Folin-Ciocalteu (F-C). The DPPH and CUPRAC methods were briefly described in Section 2.4. In the F-C method, 1.0 mL of methanolic oil extract reacted with 0.5 mL of F-C reagent for 3 min. Then, 1 mL saturated sodium carbonate solution (22.0%) was added to the reaction mixture and made up to 10 mL with redistilled water in a volumetric flask. The absorbance readings were taken at 765 nm after incubation in darkness at room temperature for 1 h. The calibration plot, $A_{765}$ = (0.0625 ± 0.0018)$c_{\mathrm{SA}}$ + (0.0947 ± 0.0144) was linear (R^2^ = 0.9958) in the concentration range between 0.88–13.20 μg/mL for methanolic solutions of sinapic acid (SA).

The absorbances were measured using a UV-Vis spectrophotometer U-2900 (Hitachi High-Technologies Corporation, Tokyo, Japan) in 1 cm glass cells at 517 nm, 450 nm, and 765 nm, respectively, for the DPPH, CUPRAC, and F-C assays. The AA was expressed as μmol of Trolox equivalents per 100 g of sample (μmol TE/100 g), while TPC was expressed as mg of sinapic acid (SA) per 100 g of sample (mg SA/100 g).

### 2.8. Fluorescence Studies of the Cold-Pressed Rapeseed Oil

Fluorescence spectra were recorded by a Gilden pλotonics fluoroSENS instrument (Gilden Photonics Ltd., Glasgow, UK) equipped with a xenon lamp and connected to a personal computer. The measurements were done using an excitation range of 250–550 nm at intervals of 5 nm and emission wavelengths from 200 to 800 nm at an interval of 5 nm. The acquisition interval and the integration time were maintained at 1 nm and 0.1 s, respectively. Fully corrected spectra were concatenated into an excitation-emission matrix (EEM). Each oil sample was put in a 10 mm quartz cuvette, transparent on all sides. Rayleigh signals were removed in all EEM by inserting the zero in regions where λem ≤ λexc and λem ≥ 2 × λexc.

SF spectra of CPRO samples diluted in n-hexane (10%) were recorded with an offset between excitation and emission monochromators of 10 and 30 nm, with a range of excitation from 200–800 nm. Monochromator slits were fixed at 1 nm, and scans were recorded with a resolution of 2 nm. Spectra were corrected for detector efficiency.

### 2.9. Statistical Analysis

The antioxidative measurements of the prepared GEL-PVA-sinapic acid ester films were performed in three replications. The oxidation (PV, p-AnV, and AV, amounts of conjugated dienes—K_232_ and trienes—K_268_) and antioxidant parameters (results of DPPH, CUPRAC, and TPC) for each oil sample were analyzed in triplicate within 1 day. The obtained results were presented as the mean (c) ± standard deviation (SD). A one-way analysis of variance (ANOVA), followed by the post hoc Duncan test, was performed to analyze the significant differences between data (*p* < 0.05).

Statistical analysis of the data was performed using the Statistica 8.0 software (StatSoft, Tulsa, OK, USA).

## 3. Results and Discussion

### 3.1. Antioxidant Properties and Thickness of Prepared Films

The proposed strips of GEL-PVA films with sinapic acid esters, in which antioxidants were loaded into GEL-PVA material to reduce the deterioration of oil-based food quality by delaying lipid oxidation, are one of the most promising additions to traditional packaging.

It is essential to use several methods to evaluate the antioxidant properties and protective effect of the amphiphilic antioxidants incorporated into the studied films. Therefore, the AA of GEL-PVA films with the three sinapic acid esters was examined using DPPH and CUPRAC methods. The CUPRAC is an assay that simultaneously examines both hydrophilic and lipophilic antioxidants, whereas the determination of hydrophilic antioxidants by the DPPH assay is limited due to the hydrophobic nature of the DPPH radical.

On the other hand, in our previous work, we observed that none of the synthesized sinapic acid derivatives added to the GEL-PVA-control film affected the viability of the model intestinal cells Caco2, whereas these esters exerted cytotoxic and anti-proliferative effects against two human cancer cell lines (HeLa and A549) [14]. Moreover, CSA and OSA had the highest ability to scavenge the DPPH radical (IC_50_ = 159.69 μmol/L) and ABTS radical cation (IC_50_ = 171.25 μmol/L), respectively. In contrast, OSA exhibited the lowest antioxidant DPPH radical scavenging activity with an IC_50_ value of 232.20 μmol/L, while ESA and CSA had a significantly lower efficiency in scavenging the ABTS radical cation (IC_50_ = 262.44 and 245.50 μmol/L) [14].

As presented in Table 1, the lowest DPPH and CUPRAC values were found for the control GEL-PVA film (DPPH = 5.62 mmol TE/100 g, CUPRAC = 4.55 mmol TE/100 g), which could be attributed to the presence of peptides with antioxidant properties in the GEL.

These properties may be attributed to the unique amino acid composition, structure, and hydrophobicity of the peptides. Depending on such features, GEL peptides could act as lipid peroxidation inhibitors, free radical scavengers, and transition metal ion chelators [35,36]. However, other authors reported that neat gelatin film had slight scavenging activities, determined by DPPH (5.1%) and ABTS (12.9%) methods [37]. In contrast, the control PVA-starch film prepared by Kumar et al. [38] had no antioxidant properties. Similarly, the pure PVA film did not exhibit any scavenging activity determined by the DPPH assay [39]. Therefore, it could be assumed that the influence of PVA on the AA of GEL-PVA films was negligible.

Most importantly, the Duncan test indicated that the addition of each sinapic acid ester resulted in a significant enhancement of the AA of the GEL-PVA films. It is noteworthy that the AA of GEL-PVA-ESA analyzed by the DPPH and CUPRAC methods increased 22-fold and 16-fold compared to the GEL-PVA film without synthesized esters. However, the DPPH and CUPRAC results for the GEL-PVA-OSA and GEL-PVA-CSA films were approximately 50% lower than the AA of GEL-PVA-ESA (Table 1). Unfortunately, the addition of CSA, with the highest ability to scavenge the DPPH radical (IC_50_ = 159.69 μmol/L), to film-forming dispersion caused the lowest increase in the AA of the fortified film GEL-PVA-CSA (DPPH = 60.20 mmol TE/100 g and CUPRAC = 36.21 mmol TE/100 g). This can be explained by the fact that 10 mL of each sinapic acid alkyl ester (ESA, OSA, and CSA) was introduced to FFS, whereas the concentration of CSA in FFS was the lowest (0.2229 mol/L), due to its high molecular weight (448.63 g/mol) [14]. The somewhat lower molecular weight of OSA (336.45 g/mol) affected its concentration in FFS (0.2972 mol/L), increasing the antioxidant potential of GEL-PVA-OSA as determined by the DPPH and CUPRAC methods (Table 1). However, ESA had the lowest molecular weight (252.26 g/mol) and resulted in the highest concentration in FFS (0.3964 mol/L) and the highest antioxidant properties of the GEL-PVA-ESA film. On the other hand, a lower increase in the antioxidant properties of the GEL-PVA films with CSA and OSA can point to the instability of these phenolipids during the preparation of the films or more interactions with the GEL-PVA matrix.

Significant differences in the concentrations of sinapic acid esters in FFS significantly influenced the AA of the prepared films (Duncan test, Table 1). It is noteworthy that the AA results of the control film measured by the DPPH and CUPRAC methods were quite similar (DPPH = 5.62 mmol TE/100 g; CUPRAC = 4.55 mmol TE/100 g). However, for films enriched with increasing alkyl chain length esters from ethyl up to cetyl, the DPPH results were almost two times higher (60.20–121.52 mmol TE/100 g) in comparison with CUPRAC values (36.21–74.25 mmol TE/100 g). These results suggest that the phenolipids present in the GEL-PVA matrix could act equally effectively as radical-free scavengers and cupric ion reducers, whereas sinapic acid esters exhibit better performance in quenching DPPH radicals.

For comparison, brown stripe red snapper skin gelatin film incorporated with other antioxidants, such as butylated-hydroxytoluene (BHT) and α-tocopherol, had a relatively low radical scavenging activity (DPPH = 0.5 and 7%, respectively) before storage, which increased after 6 weeks of storage to 3 and 14%, respectively [40]. This phenomenon could be explained by the renaturation of GEL chains into the helix coil structure during storage which leads to the release of a greater amount of antioxidants from the film. Furthermore, chicken protein isolate/fish skin gelatin blend films containing 0.75% gallic acid possessed the highest DPPH radical scavenging activity (31.22 µmol TE/g) and were used to form pouches for packing chicken skin oil. However, the DPPH value for chicken protein isolate/fish skin gelatin incorporated with 0.75% tannic acid was three times lower (11.07 µmol TE/g) [41].

The thickness of prepared films ranged from 0.183 to 0.306 mm (Table 1). It is clearly visible that the addition of sinapic acid alkyl esters resulted in a significantly (*p* < 0.05) increased thickness of the films in comparison with the GEL-PVA sample (0.183 mm). As could be noted, the carbon chain length of esters added to the control film affected their thickness, i.e., a longer chain of ester resulted in increased thickness. This effect was probably associated with the HLB and log P values of synthesized esters added to the prepared GEL-PVA films. The calculated HLB values for sinapic acid esters decreased following the order: 8.06 > 5.21 > 1.41 for ESA, OSA, and CSA, respectively. However, HLB is inversely proportional to log P; thus, ESA had the lowest log P, 2.26, CSA had the highest (log P = 8.87) [14]. Consequently, the thickness values of the prepared films significantly decreased with the increased HLB value (Duncan test, Table 1). Similar results were presented for chitosan/zein/lemon essential oil composite films and chitosan-thyme essential oil composite films [17,19]. The thickness of these composite films decreased along with the increase in HLB values of various emulsifying agents. This was explained by the fact that emulsifiers, as amphiphilic substances, are able to reduce interactions between the chitosan and essential oil molecules and cause a decrease in film thickness. The opposite effect was observed for agar/maltodextrin-beeswax emulsion films, where higher thickness values (51.00 mm, 50.67 mm) were observed in films with emulsifiers with higher HLB values (8.3, 15.0) [20].

On the other hand, the increased thickness of the prepared films might be related to the fact that particular esters have not been fully solubilized in FFS and could be embedded on the surface of the films. For comparison, gelatin/polyethylene bilayer films with a higher concentration (9%) of fruit peels demonstrated increased thickness (0.20, 0.18, and 0.25 mm for films incorporated with pomegranate, papaya, and jackfruit peels, respectively) compared to the control film (0.12 mm), due to embedded fruit particles on the film surfaces [42]. Such film thickness variations could affect the release rate of active substances from the prepared films to food simulants. Additionally, these authors observed an increase in the radical scavenging activity of films loaded with fruit peel powders determined by the DPPH and ABTS methods and their thickness with increasing concentrations of added powders from 1 to 9%. Similarly, fish gelatin films with mango kernel extract demonstrated increased antioxidant properties (DPPH = 5, 22, 45, and 60%, for the control film and films with 1, 3, and 5% of the extract, respectively) with the increasing thickness (0.055, 0.064, 0.076, and 0.090 mm for the control film and films with 1, 3, and 5% of the extract, respectively) [43].

Meanwhile, Mastromatteo et al. [44] found that film thickness could accelerate or delay the thymol release rate. Moreover, it was reported that increased film thickness resulted in a more intense migration of added substances from the film edge [45]. Therefore, the thickness of the prepared GEL films strongly depends on the preparation method and the addition of other macromolecules such as PVA and sinapic acid esters with different HLB values.

### 3.2. Effect of Active Films on the Antioxidant Activity of Cold-Pressed Rapeseed Oil during Accelerated Storage

The effectiveness of GEL-PVA films incorporating sinapic acid esters as new antioxidant packaging systems was tested for CPRO samples packaged in amber glass bottles stored at accelerated conditions for two weeks.

The AA and TPC in CPRO samples without and with active film strips after 7 and 14 days of storage at 40 °C under light were determined by the modified DPPH, CUPRAC, and F-C methods, and the obtained results are presented in Table 2.

The Duncan test indicated that the AA and TPC in the studied oil samples analyzed during 2 weeks of accelerated storage by the three proposed analytical assays differ from each other. The different mechanisms of the analytical methods used probably affected these discrepancies between the AA results. As can be seen, the CUPRAC values of all oil samples were significantly higher than the DPPH results. This can be explained due to the fact that the CUPRAC assay can be applied to fat-based matrices containing both lipophilic and hydrophilic antioxidants. The CUPRAC method is based on the single electron transfer (SET) mechanism and measures the ability of antioxidants to transfer one electron for the reduction of cupric (Cu^2+^) to cuprous (Cu^+^), which forms colored complexes with ligands (commonly with neocuproine). However, the DPPH values for all investigated oil samples were approximately 4–8.5 times lower in comparison with CUPRAC results (Table 2). It is known that the DPPH test is based on the measurement of the scavenging activity of antioxidants present in methanolic oil extracts toward colored DPPH radicals. This analytical method is a mixed-mode test where SET, hydrogen atom transfer (HAT), and proton-coupled electron transfer (PCET) mechanisms may play different roles in varied proportions.

It can be noted that the CPRO packed in the original amber glass marasca bottles had the lowest DPPH (345.19 µmol TE/100 g), CUPRAC (1929.02 µmol TE/100 g), and TPC (3.02 mg SA/100 g) results after 2 weeks of the accelerated storage, indicating degradation of antioxidants naturally present in the oil (Table 2). Insignificantly higher values of AA and TPC were found for CPRO kept in a closed bottle over 7 days of accelerated storage. However, the antioxidant potential of fresh CPRO was significantly higher than the DPPH and CUPRAC of stored samples (Duncan test, Table 2). The AA and TPC values in CPRO samples stored in bottles with prepared active antioxidant film strips for 7 and 14 days were higher than the antioxidant potential of fresh oil. Still, a significant decrease in the antioxidant properties of oil samples was detected up to the end of the study (Duncan test, Table 2). Nevertheless, the one-week storage of CPRO samples with GEL-PVA-sinapic acid esters at 40 °C under a fluorescent lamp caused an approximately 1.2–2.1, 1.1–1.4, and 1.6–14.0 times increase in the DPPH, CUPRAC, and TPC values, respectively. This can be explained by the fact that sinapic acid esters as amphiphilic antioxidants purposefully added to films were released from the biopolymeric active strips into the stored oils, enhancing their antioxidant properties. Unexpectedly, the GEL-PVA strips without sinapic acid esters immersed in CPRO samples also increased their AA and TPC. It is likely the biopolymeric material had antioxidant potential, which could be transferred into the oil, and these bioactive compounds caused a synergic effect with antioxidants naturally occurring in the CPRO. As expected, the AA and TPC in the CPRO samples increased with the increasing antioxidant properties of the added film strips (Table 1 and Table 2). The highest antioxidant potential was in oil with GEL-PVA-ESA, having the highest DPPH and CUPRAC results, whereas oil with control strips of GEL-PVA revealed the lowest DPPH, CUPRAC, and TPC values.

However, the AA and TPC results of the studied oil samples demonstrated a significant decrease in antioxidant power throughout the storage time in the following order CPRO + GEL-PVA-ESA > CPRO + GEL-PVA-OSA > CPRO + GEL-PVA-CSA > CPRO + GEL-PVA > CPRO (Duncan test, Table 2). After storage, the antioxidant compounds in oils disappeared because oxidation reactions and decomposition caused the loss of these bioactive compounds, which depend on their chemical structures, storage conditions, additives, and types of containers [46].

Interestingly, insignificant differences were observed between the DPPH and CUPRAC values for fresh CPRO and oil containing strips of GEL-PVA-CSA after accelerated storage for 14 days (Duncan test, Table 2). Moreover, the same DPPH values were found for CPRO + GEL-PVA-OSA and CPRO + GEL-PVA-ESA during storage for 7 and 14 days, respectively. The Duncan test indicated that CPRO samples with GEL-PVA-OSA and GEL-PVA-CSA after 7 days of storage and the CPRO with control strips (GEL-PVA) after 14 days did not differ significantly in CUPRAC results. Unexpectedly, total amounts of phenolic compounds in fresh CPRO (4.23 mg SA/100 g) had reduced insignificantly during the first (3.53 mg SA/100 g) and the second week of storage (3.02 mg SA/100 g). Moreover, the control GEL-PVA film strips did not significantly affect the total polyphenols concentration in CPRO samples stored in accelerated conditions for the two periods studied (Duncan test, Table 2). Although, insignificantly higher TPC was determined in CPRO + GEL-PVA-CSA than in CPRO + GEL-PVA after 14 and 7 days of storage, respectively.

These results confirm again the antioxidant properties already measured in the active biopolymer materials loaded with sinapic acid esters. Sinapic acid esters added to the GEL-PVA films had significant antioxidant potential, and their release into CPRO samples created an effective defense system against free radical attack through the ability to break the free radical chain reaction by donating H-atom(s) from hydroxyl and/or methoxy groups in an aromatic ring [47].

On the other hand, our previous work demonstrated that crude oils pressed from rapeseed stored in gelatin sachets incorporated with rapeseed meal had higher antioxidant potential than an oil pressed from fresh rapeseed. This suggested that antioxidants released from the bag significantly affected the content of antioxidants in the packed rapeseed, leading to an increase in the antioxidant properties of the pressed oil [25]. The migration process of natural antioxidants was probably slower than in the case of sinapic acid esters due to greater bonding affinity between the carboxylic group from hydrophilic phenolic acid and amine groups of GEL.

Most recent research has focused on the antioxidant properties of active packaging, determined by different analytical methods with very limited reports of the AA of edible oils and oil-based products stored in these active antioxidant materials [41,48,49,50,51].

For comparison, the transparent plastic film loaded with a metalized material caused the highest TPC reduction (52–64%) in two Sardinian extra-virgin olive oils after storage at 40 °C and 60 °C for 96 and 32 days, respectively. However, the best-performing material in protecting olive oil phenolic content was the transparent plastic packaging incorporating a UV-blocker (TPC reduction = 21–30%), while olive oils stored in the brown-amber glass revealed somewhat lower phenolic levels (TPC reduction = 29–40%) [52]. Moreover, Jamróz et al. [48] observed the best protective properties of furcellaran film incorporated with graphene oxide against the degradation of total carotenoids in cold-pressed linseed oil subjected to UV-B treatment (583.9 and 572.9 mg/kg carotenoid amounts in untreated oil and treated oil, respectively, placed on a Petri dish covered completely with film).

In addition, the nature of the packaging material had a notable influence on the phenolic content in Chemlali extra-virgin olive oil after 180 days of storage [46]. The highest decrease in phenolic content was observed for olive oils stored in polyethylene containers (from 363 to 193 mg/kg) and clear glass bottles (from 363 to 206 mg/kg). However, the loss of TPC was significantly lower (20–25%) in olive oils stored in a tin container and dark bottle. This fact indicated the joint action of light and the permeability of the polyethylene container to the oxygen that catalyzes the oxidation reaction.

### 3.3. Effect of Active Films on the Oxidative Status of Cold-Pressed Rapeseed Oil during Accelerated Storage

The effect of accelerated storage on the oxidative status of CPRO samples without and with film strips incorporating different sinapic acid esters was evaluated using characteristic parameters such as PV, p-AnV, K_232_, K_268_, TOTOX, and AV associated with primary and secondary oxidation products as well as free fatty acids. As presented in Table 3, all oil samples demonstrated an increase in their results of PV, p-AnV, TOTOX, and AV during storage.

The PV was used to measure primary oxidation products. Therefore, the PV presents the susceptibility of oils to oxidation by measuring peroxides and hydroperoxides and could be used as an indicator for the initiation of oxidation. The initial PV of the CPRO was 2.85 meq O_2_/kg. It can be noted that the PV results of CPRO samples kept closed in original bottles and contacted with different film strips during the whole storage process were below the maximum value (15 meq O_2_/kg of oil) reported in the ISO 3960 (2017) [31] for cold-pressed vegetable oils. The Duncan test indicated insignificant differences between the PV results for CPRO samples with control GEL-PVA and GEL-PVA-ESA and GEL-PVA-CSA films (Table 3). Moreover, similar amounts of hydroperoxides in fresh CPRO and oil stored for 7 days under accelerated conditions in the presence of GEL-PVA-OSA were observed. In contrast, the highest increase in PV, from 2.85 to 7.04 meq O_2_/kg, after two weeks of storage was found for commercial CPRO samples stored under accelerated conditions. Therefore, the delay in the oxidation process of CPRO samples could be due to the antioxidant properties of strips loaded with antioxidants, with the sinapic acid esters having antioxidant potential determined by three analytical methods (Table 2). However, the various antioxidants added to the film strips showed varying efficacy in inhibiting hydroperoxide formation. Nevertheless, these primary oxidation products can be further decomposed into non-volatile and volatile secondary products such as carbonyl compounds, including aldehydes and ketones. Therefore, the p-AnV has a major role with respect to the oxidation of vegetable oils and provides useful information on carbonyl compounds, which are important contributors to the off-flavors associated with the rancidity of many oil-based products.

As can be seen, the p-AnV results listed in Table 3 significantly increased in all studied CPRO samples (p-AnV = 1.64–5.90) during storage when compared to fresh CPRO (p-AnV = 0.46). After 7 and 14 days of accelerated storage, the p-AnV results (4.74 and 5.90, respectively) were the highest for the CPRO packed in standard dark bottles. Interestingly, the addition of control GEL-PVA film strips without sinapic acid esters to CPRO retarded the formation of secondary oxidation products with a p-AnV of 3.62 and 5.51 on the 7th and 14th day of storage, respectively. However, CPRO + GEL-PVA-ESA revealed the lowest increase in p-AnV (1.64 and 3.33), followed by oil samples with GEL-PVA-OSA (p-AnV = 2.55 and 3.37) and GEL-PVA-CSA (p-AnV = 2.85 and 4.13 on the first and second week of storage, respectively). This can be explained by the fact that the antioxidant potential of films was the highest after being enriched with ESA, followed by OSA, and the lowest for GEL-PVA strips loaded with CSA (Table 1). Therefore, the slowest rate of secondary oxidation was perceived in an oil sample containing GEL-PVA-ESA with the highest antioxidant properties determined using three assays, namely, DPPH, CUPRAC, and F-C. However, insignificant differences in p-AnV results were observed between oil with control film strips on the 7th day of storage and oil samples stored during 14 days in the presence of film strips incorporating ESA and OSA (Duncan test, Table 3). Thus, the p-AnV results increased as the storage time increased but the number of secondary oxidation products was less in oil samples with enriched film strips. The lower PV for CPRO + GEL-PVA showed an unexpected trend. In contrast, the p-AnV results for oil samples containing GEL-PVA film strips with sinapic acid esters were lower than the p-AnV for CPRO + GEL-PVA, indicating the antioxidative efficiency of these esters in slowing down the oxidation rate and controlling oxidative stability. This suggests that the simultaneous measurement of primary and secondary oxidation products is essential to assure effective monitoring of the CPRO oxidation advancement. For this reason, total oxidation values (TOTOX) combining the amounts of primary with secondary oxidation products were applied to estimate the oxidative deterioration of the analyzed CPRO samples under accelerated storage conditions. It is noteworthy that the presence of film strips with sinapic acid esters in the studied oil samples caused a decrease in their TOTOX indexes (Table 3). The highest TOTOX values were observed for oil samples stored during 7 and 14 days (13.06 and 19.98, respectively) under accelerated conditions in typical bottles without film strips. However, lower TOTOX results (6.16–19.98) for all investigated CPRO samples than that recommended by the German Guidelines for Edible Fats and Oils (<20) suggest their high quality in terms of oxidative rancidity [53].

Furthermore, K_232_ and K_268_ associated with conjugated dienes and conjugated trienes, respectively, are suitable parameters for the evaluation of the oxidative deterioration of the oil. The absorbance at 232 nm (K_232_) gives information about the presence of diene conjugates, while the specific coefficient of extinction at 268 nm (K_268_) measures secondary oxidation products (aldehydes and ketones) produced by the breakdown of hydroperoxides. Interestingly, oil samples containing GEL-PVA film strips with sinapic acid esters showed a high rate of primary oxidation and delayed secondary oxidation, giving higher K_232_ and lower K_268_ values (Table 3). It can be noted that the K_232_ values for fresh CPRO and stored CPRO with film strips incorporating OSA and CSA did not change significantly during the accelerated storage period, while significantly higher amounts of conjugated diene (K_232_ = 1.894–2.014) were determined in CPRO samples without and with film strips containing ESA at the end of the 7 and 14 days of storage (Duncan test, Table 3). Unexpectedly, CPRO + GEL-PVA revealed lower double bond conjugation concentrations than oil samples with the prepared strips, rich in antioxidants during the accelerated oxidation process. The higher values observed in these oil samples could be linked with the evolution of peroxides observed in fresh oil (K_232_ = 1.746), which formed during the bottling phase. On the contrary, in regard to K_268_, CPRO + GEL-PVA showed an absorption (0.215 and 0.257 after 7 and 14 days, respectively) that was higher than the others (0.142–0.160 and 0.152–0.205), while the differences between amounts of conjugated trienes in the oil samples containing film strips loaded with sinapic acid esters at the same storage week were not statistically significant (Duncan test, Table 3). However, a significant increase in conjugated trienes (K_268_ = 0.291 and 0.310) was observed during accelerated storage in the case of the CPRO packed in its original dark bottles. The lower values of K_268_ in the CPRO samples with fortified film strips could be due to the partial degradation of the trienes formed in the oil samples during storage conditions, up to 14 days, caused by the ability of sinapic acid esters in the film strips to inhibit the formation of conjugated bonds. On the other hand, during accelerated storage conditions, free radicals attack the double bond of unsaturated fatty acids, forming a conjugated bond even if some of the double bonds were destroyed during autoxidation. In addition, volatile compounds can be generated.

For comparison, the increase in primary and secondary oxidation products determined as PV, K_232_, K_268,_ and p-AnV during the accelerated storage of oils in active packages from biopolymers with the combination of the phenolic compounds was observed in our previous work and by other authors [24,25,52,54,55]. Crude rapeseed oils pressed from seeds in gelatin sachets without and with rapeseed meal after 7 and 14 days of storage had significantly higher PV (0.68–0.79 meq O_2_/kg) and p-AnV (0.66–1.01) than the PV (0.56 meq O_2_/kg) and p-AnV (0.49) for oil pressed from fresh rapeseed (before packaging) [25].

Moreover, plant-based guar gum fibrous mats encapsulating tannic acid as a natural antioxidant were used to improve the oxidative resistance of unrefined flaxseed oil during storage. The tannic acid fibrous mat afforded the highest protection from oxidation after 30 days of storage at 60 °C. The PV (6–14 meq O_2_/kg) and p-AnV (10–25) results for oils with the tannic acid fibrous mat were lower than those of the control oils (PV = 11–17 meq O_2_/kg, p-AnV = 20–65) during 30 days of accelerated storage. The encapsulated tannic acid significantly inhibited the formation of conjugated dienes in the flaxseed oil, and an increase of 33% was observed between 0 and 30 days. The added tannic acid fibrous mat was an effective antioxidant almost every day of storage, indicating that the natural antioxidants encapsulated in this nanoscale delivery system could successfully delay both the primary and secondary oxidation reactions of flaxseed oil throughout the tested storage time [24].

Similarly, the lowest amounts of peroxides (PV results were approximately 1 meq O_2_/kg) and conjugate dienes (about 7 mmol/L) were observed in soybean oils containing the nanoencapsulated extracts of Iranian golpar with a wall of sage seed gum and chitosan after storage at room temperature for 30, 45, and 60 days. Although, fewer primary oxidation products were found in oil samples containing free extract on 1 and 15 days of storage than in oil samples containing the nanoencapsulated extract [54]. In addition, a Pistacia khinjuk extract nanoemulsion in the biopolymeric coating showed promising results for the retardation of primary (PV = 2.24–3.17 meq O_2_/kg) and secondary (p-AnV = 3.54–4.67) oxidation product formation in comparison with the control sample (PV = 2.24–5.73 meq O_2_/kg and p-AnV = 3.54–5.31) in sunflower oils stored at 60 °C for 24 days [55].

In contrast, after an initial slight PV increase in two Sardinian monovarietal olive oils stored at 40 and 60 °C in two innovative packaging materials for 96 and 32 days, respectively, a decrease was observed [52]. Olive oils stored in 100% compostable metalized bio-based material had lower PV results than peroxide amounts in olive oil samples packed in transparent plastic film from low-density polyethylene loaded with a UV-blocker (polyethylene terephthalate). The PV decreased with the appearance of secondary oxidation products in olive oils; thus, the K_270_ parameter as a good marker of the secondary oxidation stage significantly increased during the accelerated test. However, the extinction at 270 nm at the end of storage was higher for olive oil in metalized material in comparison with olive oil packed in transparent plastic material.

On the other hand, AV could be used as a quality oil index during storage. It is well known that a lower AV represents the higher stability of vegetable oils. It is noteworthy that the AV initial value of fresh CPRO was the lowest (Table 3). However, the formation of free acids in this oil during storage was the most intense, and AV results reached 0.51 and 0.63 mg NaOH/g after 1 and 2 weeks, respectively. The storage time under accelerated conditions caused a gradual and significant increase in the acidity of all oil samples (Duncan test, Table 3). Nevertheless, the AV results (0.30–0.63 mg NaOH/g) of studied oil samples during the whole storage process were below the maximum values (AV ≤ 4 mg NaOH/kg) permitted for vegetable oils according to the ISO 660 (2009) [33]. Interestingly, the GEL-PVA films incorporating sinapic acid esters produced a somewhat higher increase in the acidity of CPRO samples. These antioxidant compounds were likely released from film to oil samples and increased their AV results. As can be seen, the addition of film strips fortified with ESA ester to glass bottles appeared to be a package in which the inhibition of the hydrolytic processes of the glycerides was less effective than in oil samples containing other dipped polymer film strips (AV = 0.47 and 0.50 mg NaOH/g for CPRO + GEL-PVA-ESA stored 7 and 14 days, respectively, in the accelerated conditions). Unexpectedly, no significant variations were observed during the 1st and 2nd-week storage of CPRO samples with control film and samples with films loaded with OSA and CSA (Duncan test, Table 3). This fact indicates that the free acidity of CPRO samples in the presence of the added film strips did not undergo significant changes over accelerated storage, remaining stable for up to 2 weeks.

Recently, innovative solutions such as nanoencapsulation and new packaging materials have caused the progressive acidity of sunflower, soybean, and olive oils after storage under different conditions as a consequence of the hydrolysis of triglycerides and the production of carbonyl groups during the autoxidation process [52,54,55]. Although, the sunflower and soybean oils containing the Pistacia khinjuk extract nanoemulsion in a biopolymeric coating and the Iranian golpar extract nanoencapsulated with chitosan and sage seed gum demonstrated lower AV values (0.18–0.34 and about 0.3 mg KOH/g, respectively) compared to the AV for the control sunflower (0.16–1.18 mg KOH/g) and soybean (0.3–1.5 mg KOH/g) oils [54,55]. However, at 40 °C, a similar AV increase was observed for olive oils stored in a transparent plastic film loaded with a UV-blocker and metalized material, whereas at 60 °C, the lowest acidity was seen for olive oils stored in metalized packaging [52].

### 3.4. Fluorescence Studies of Cold-Pressed Rapeseed Oil during Accelerated Storage

**Figure 3:**
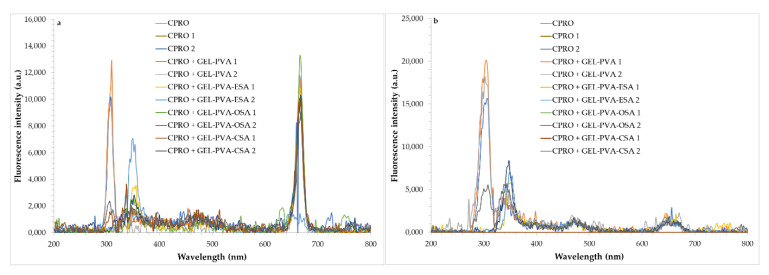
Synchronous fluorescence spectra of cold-pressed rapeseed oil (CPRO) samples packaged in dark glass bottles without and with film strips diluted in n-hexane (c = 10%) and recorded at Δλ = 10 nm (**a**) and Δλ = 30 nm (**b**) after storage at accelerated conditions.

Synchronous fluorescence (SF) spectroscopy was applied to analyze the oxidation rate of CPRO samples packaged in original bottles with immersed film strips incorporating sinapic acid esters under accelerated storage conditions (Figure 3). The two intervals (Δλ = 10 and 30 nm) between excitation and emission wavelengths were chosen because Δλ in SF spectra determines the shapes, bandwidths, and fluorescence intensities. In this way, spectra selectivity is increased, and the fluorophore components present in CPRO samples during oxidation processes can be better characterized.

As shown in the SF spectra (Figure 3a,b), there are apparent differences between 640 nm and 680 nm due to the chlorophyll derivatives present in the CPRO samples [56]. It was found that at Δλ = 10 nm, the SF spectra of the CPRO samples had better-resolved features for chlorophyll derivatives, and bands caused by tocopherols and exogenous antioxidants were better separated, while at Δλ = 30 nm, SF spectra had better-resolved features for oxidation products. However, after the oil samples were stored, the fluorescence maxima of the bands changed. Figure 3 demonstrates the fluorescence maximum of the emission band at 310 nm ascribed to tocopherols (vitamin E). It is evident from Figure 3 that this peak decreased and moved after the addition of the film strips with sinapic acid esters to oils. The fluorescence intensity was the lowest for CPRO + GEL-PVA-CSA stored under accelerated conditions for 7 and 14 days. The CPRO samples with enriched film strips had strong bands in a region between 310–365 nm, attributed to phenolic compounds [47]. The intensities of these bands depended on the film type immersed in oil. Thus, the maximal intensity appeared for oil containing ESA, with the highest antioxidant potential determined by three different analytical methods (Figure 3 and Table 2).

Interestingly, the emission bands between 400 and 550 nm caused by the generation of oxidation products had low intensities for each studied oil during the storage period. This can be explained by the fact that all analyzed oxidative parameters, such as PV, p-AnV, K_232_, K_268,_ and AV, were below the legal limits (Table 3).

It is noteworthy that the fluorescence maximum assigned to chlorophyll at 668 nm was enhanced in oil samples with film strips (Figure 3a). The peak at 668 nm of control CPRO without film strips had the lowest intensity. Although, the peak at 668 nm of oil samples with film strips became much stronger and this band evolved to lower intensity values after storage in accelerated conditions. This phenomenon occurs due to the decreased chlorophyll concentration in the oils after storage.

It was found that at Δλ = 30 nm (Figure 3b), the SF spectra of CPRO samples had better-resolved features but shifts and changes in the fluorescence maxima at 305, 345, 470, and 650 nm, characteristic for tocopherols, phenolic compounds, oxidation products, and chlorophyll, were observed during accelerated storage up to 2 weeks at 40 °C under a light. As can be seen, phenolic antioxidants were also present in the control samples, although the intensities of these bands ranged between 340–365 nm and were lower for control CPRO without film strips than for oil samples containing film strips loaded with sinapic acid esters (Figure 3). However, the synchronous spectra for higher Δλ = 30 nm revealed a weak band with a maximum at about 650 nm, which can be attributed to chlorophyll derivatives (Figure 3b). In contrast, the oxidation products showed a stronger fluorescence peak at about 470 nm for Δλ = 30 (Figure 3b).

Other authors also used SF spectroscopy to characterize and evaluate the quality of various rapeseed oils based on their degree of oxidation [47,56,57]. In our previous work, the addition of synthesized phenolic acid derivatives to refined rapeseed oils enhanced the intensities of bands characteristic of phenolic compounds. However, the intensities of these bands decreased after the storage of oil samples under different conditions [47]. In contrast, the SF spectra recorded at Δλ = 40 for rapeseed oil during heating demonstrated two strong peaks from 590 to 700 nm and five weak peaks from 330 to 590 nm [57]. The band with the highest intensity at 362 nm was ascribed to the decrease in antioxidants due to heating, but the changes in the bands from chlorophyll and its derivatives at 590–700 nm were small due to the high thermal stability of these compounds. The fluorescence peak intensities on the SF of rapeseed oil changed slightly at 60 and 130 °C, while a new characteristic peak replaced the original weak peaks at 190 °C due to the formation of oxidation products from unsaturated fatty acids. Moreover, to evaluate the three types of canola oils (cold pressed, chemically extracted, and eight commercial brands), their emission spectra were recorded by using excitation wavelengths from 280–420 nm with a step of 10 nm [56]. The emission bands at 375, 525, 673, and 725 nm, which were common in oils extracted chemically and cold-pressed and absent in the eight commercial brands, consequently, lead to the conclusion that the commercially available oils did not contain tocopherols, carotenoids, and chlorophylls. Nevertheless, up to 180 °C, no appreciable spectral changes occurred in the cold-pressed canola oil samples. Above 180 °C, the number of oxidative products increased, and thus, the intensities of bands at 375 and 673 nm decreased because the essential fatty acids and fat-soluble vitamins, carotenoids, and chlorophyll were oxidized. In contrast, the intensity of the emission band at 525 nm, representing vitamin E and β-carotene, increased with the rise in temperature above 150 °C, possibly due to the oxidation of these nutritional ingredients.

**Figure 4:**
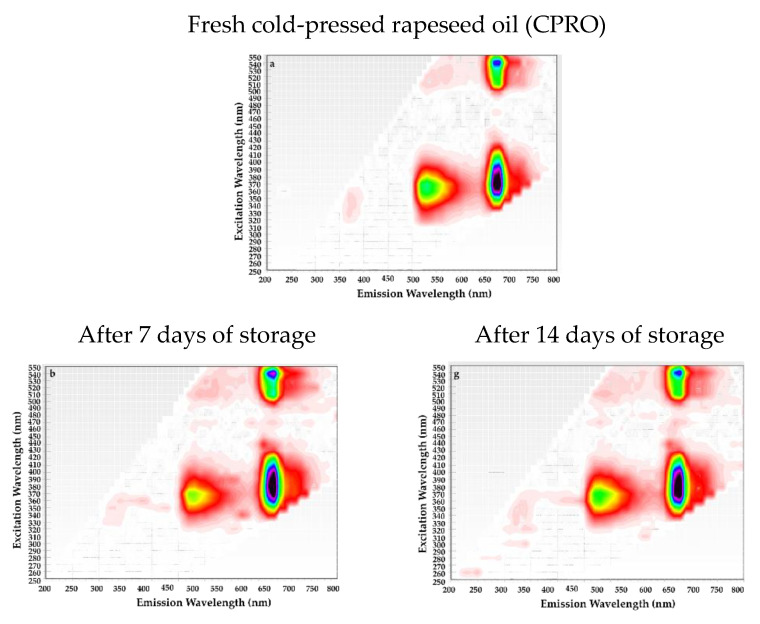

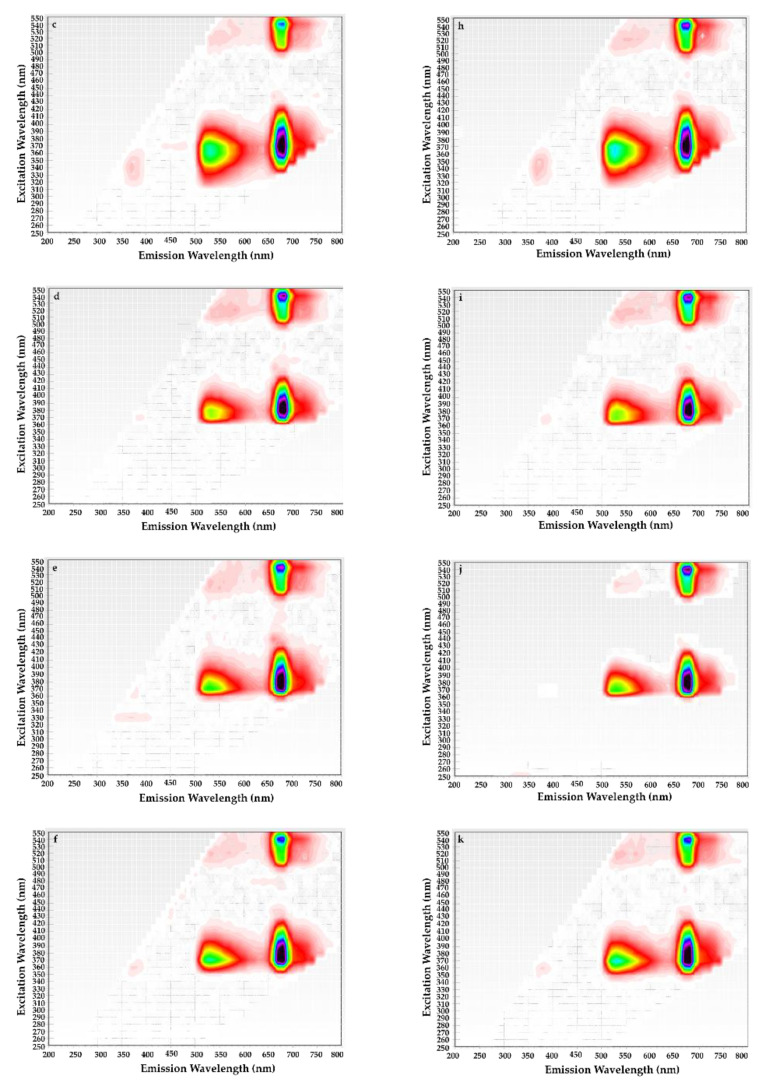
Excitation-emission matrices of fresh cold-pressed rapeseed oil (CPRO) (**a**), CPRO samples without and with film strips after 7 days (**b**–**f**) and 14 days (**g**–**k**) of storage: CRPO (**b**,**g**), CPRO + GEL-PVA (**c**,**h**), CPRO + GEL-PVA-ESA (**d**,**i**), CPRO + GEL-PVA-OSA (**e**,**j**), and CPRO + GEL-PVA-CSA (**f**,**k**).

Additionally, the two-dimensional excitation-emission matrix (EEM) fluorescence spectroscopy of CPRO samples without and with film strips incorporating sinapic acid esters depicted the changes in the excitation and emission profiles of the fluorescent components in each oil over a period of 2 weeks (Figure 4).

As can be seen from Figure 4a, there are three short- and long-wavelength regions for EEM of fresh CPRO, region I (λexc/λem ca. 340/370 nm), region II (λexc/λem ca. 370/525 nm), and region III (λexc/λem ca. 400 and 540/680 nm), closely associated with tocopherols, phenolic compounds, and chlorophyll and pheophytin, respectively. However, the intensity of these fluorescence bands changed considerably in the stored CPRO samples without (Figure 4b,g) and with film strips (Figure 4c–k). The addition of film strips with sinapic acid esters to CPRO samples subjected to storage time caused a decrease and disappearance in the fluorescent band in the short wavelength region (λexc/λem = 340/370 nm) corresponding to tocopherols. This phenomenon can be explained by the fact that released antioxidants from the film strips consumed naturally present tocopherols in CPRO samples. In general, the fluorescence intensity of the emission wavelength of 525 nm, belonging to antioxidants, gradually reduced with storage time. However, the intense fluorescent bands in the long wavelength region (λexc/λem = 400 and 540/680 nm), corresponding to chlorophyll and their derivatives, remained almost stable during the storage of all CPRO samples (Figure 4). This suggests that storage at 40 °C under light for 2 weeks did not cause the degradation of chlorophylls in the studied oil samples. However, the storage time affected the formation of oxidation products in CPRO samples. Two CPRO samples without film strips during the storage period (Figure 4b,g) and the CPRO+GEL-PVA (Figure 4c) stored for 7 days at accelerated conditions showed a weak emission band with the excitation maximum at 370 nm and the emission maximum at 450 nm. The formation of oxidation products may be responsible for the fluorescence at emission wavelengths between 380 nm and 500 nm. During two weeks of storage, the EEM matrices for oil samples with fortified film strips revealed a lack of emission bands between 380 and 500 nm. Generally, the EEM spectra of the investigated CPRO samples did not demonstrate significant differences between the first and second week of storage.

For comparison, Sikorska et al. reported differences in the shape and intensity of the fluorescence bands characteristic of tocopherol and chlorophyll/pheophytin of EEM spectra between fresh cold-pressed rapeseed oil and samples stored for six months, exposed to light in colorless and green bottles and stored in darkness [2]. However, the contour plots of the rapeseed oil depicted an increase in fluorescence intensity from 420 to 460 nm at 190 °C, produced from the oxidation products, while changes in intensity at 60 and 130 °C were undetected [57].

## 4. Conclusions

New GEL-PVA films incorporated with sinapic acid alkyl esters were successfully prepared. The fortified film strips significantly enhanced the antioxidant potential of CPRO samples stored for two weeks under accelerated conditions. Moreover, oil samples containing film strips loaded with amphiphilic antioxidants were identified as more oxidatively stable than control oil packed in the original dark bottle, indicating the high antioxidant potential of released sinapic acid esters against primary and secondary oxidation products after two weeks of accelerated storage. In addition, the fluorescence of fluorophores such as tocopherols, phenolic compounds, chlorophyll derivatives, and oxidation products in fresh and aged CPRO samples without and with film strips was investigated. The typical excitation and emission wavelengths for fluorophores were observed on SF and EEM spectra. The obtained results indicated that fluorescence spectroscopy could be applied for the rapid and non-destructive monitoring of the antioxidant effect of immersed film strips during the storage of CPRO samples under accelerated conditions. Based on these findings, the proposed films appear to be promising in extending the shelf life of cold-pressed vegetable oils.

## Figures and Tables

**Table 1:** Antioxidant activity and thickness of the prepared films.

Film Type	DPPH * ± SD (mmol TE/100 g)	CUPRAC * ± SD (mmol TE/100 g)	Thickness ** ± SD (mm)
GEL-PVA	5.62 ± 0.26 ^a^	4.55 ± 0.14 ^a^	0.183 ± 0.003 ^a^
GEL-PVA-ESA	121.52 ± 1.96 ^d^	74.25 ± 1.71 ^d^	0.230 ± 0.005 ^b^
GEL-PVA-OSA	62.94 ± 0.38 ^c^	39.28 ± 1.46 ^c^	0.255 ± 0.008 ^c^
GEL-PVA-CSA	60.20 ± 0.67 ^b^	36.21 ± 0.90 ^b^	0.306 ± 0.012 ^d^

* *n* = 3; ** *n* = 10; SD—Standard Deviation; Different letters (a–d) within the same column indicate significant differences between parameters of the studied films (one-way ANOVA and Duncan test, *p* < 0.05).

**Table 2:** Changes in the antioxidant properties of cold-pressed rapeseed oil without and with GEL-PVA film strips loaded with sinapic acid esters during storage for two weeks at accelerated conditions (T = 40 °C, fluorescent lamp).

Storage Time (Week)	Sample	Antioxidant Properties		
		DPPH * ± SD (µmol TE/100 g)	CUPRAC * ± SD (µmol TE/100 g)	TPC * ± SD (mg SA/100 g)
0	CPRO	389.44 ± 8.08 ^c^	2342.43 ± 110.21 ^b^	4.23 ± 0.21 ^a^
	CPRO	351.33 ± 4.11 ^a^	1945.70 ± 84.54 ^a^	3.53 ± 0.08 ^a^
1	CPRO + GEL-PVA	454.03 ± 19.39 ^d^	2567.41 ± 74.80 ^c^	6.59 ± 0.25 ^b,c^
	CPRO + GEL-PVA-ESA	835.76 ± 9.63 ^h^	3323.15 ± 166.36 ^f^	59.23 ± 2.04 ^h^
	CPRO + GEL-PVA-OSA	621.29 ± 28.07 ^g^	3097.63 ± 142.91 ^e^	30.30 ± 0.85 ^f^
	CPRO + GEL-PVA-CSA	489.42 ± 11.98 ^e^	2962.87 ± 136.75 ^d,e^	11.59 ± 0.49 ^d^
	CPRO	345.19 ± 4.98 ^a^	1929.02 ± 95.11 ^a^	3.02 ± 0.10 ^a^
2	CPRO + GEL-PVA	358.10 ± 17.29 ^a,b^	3017.26 ± 110.81 ^e^	5.80 ± 0.30 ^b^
	CPRO + GEL-PVA-ESA	621.39 ± 30.03 ^g^	3327.21 ± 129.18 ^f^	49.18 ± 2.29 ^g^
	CPRO + GEL-PVA-OSA	550.14 ± 4.94 ^f^	2820.43 ± 135.46 ^d^	23.03 ± 0.77 ^e^
	CPRO + GEL-PVA-CSA	380.37 ± 17.85 ^b,c^	2187.82 ± 71.46 ^b^	7.73 ± 0.32 ^c^

* *n* = 3; SD—Standard Deviation; Different letters (a–h) within the same column indicate significant differences between antioxidant properties of the stored oil samples (one-way ANOVA and Duncan test, *p* < 0.05).

**Table 3:** Changes in the oxidative status of cold-pressed rapeseed oil without and with GEL-PVA film strips loaded with sinapic acid esters during storage for two weeks at accelerated conditions (T = 40 °C, fluorescent lamp).

Storage Time (Week)	Sample	PV * ± SD (meq O_2_/kg)	pAnV * ± SD (-)	TOTOX (-)	K_232_ * ± SD (a.u.)	K_268_ * ± SD (a.u.)	AV * ± SD (mg NaOH/g)
0	CPRO	2.85 ± 0.17 ^a^	0.46 ± 0.02 ^a^	6.16	1.746 ± 0.052 ^b^	0.154 ± 0.002 ^a^	0.30 ± 0.01 ^a^
	CPRO	4.16 ± 0.20 ^d^	4.74 ± 0.18 ^f^	13.06	1.977 ± 0.025 ^c^	0.291 ± 0.014 ^d^	0.51 ± 0.02 ^e^
1	CPRO + GEL-PVA	3.25 ± 0.16 ^b^	3.62 ± 0.06 ^d^	10.12	1.606 ± 0.070 ^a^	0.215 ± 0.010 ^b^	0.37 ± 0.02 ^b^
	CPRO + GEL-PVA-ESA	3.37 ± 0.14 ^b,c^	1.64 ± 0.02 ^b^	8.38	1.995 ± 0.101 ^c^	0.142 ± 0.002 ^a^	0.47 ± 0.02 ^d^
	CPRO + GEL-PVA-OSA	2.97 ± 0.17 ^a^	2.55 ± 0.15 ^c^	8.49	1.753 ± 0.030 ^b^	0.160 ± 0.006 ^a^	0.42 ± 0.01 ^c^
	CPRO + GEL-PVA-CSA	3.50 ± 0.06 ^c^	2.85 ± 0.12 ^c^	9.85	1.759 ± 0.061 ^b^	0.152 ± 0.005 ^a^	0.38 ± 0.00 ^b^
	CPRO	7.04 ± 0.28 ^e^	5.90 ± 0.06 ^h^	19.98	2.014 ± 0.107 ^c^	0.310 ± 0.012 ^d^	0.63 ± 0.03 ^f^
2	CPRO + GEL-PVA	3.52 ± 0.16 ^c^	5.51 ± 0.09 ^g^	12.55	1.657 ± 0.008 ^a,b^	0.257 ± 0.008 ^c^	0.42 ± 0.01 ^c^
	CPRO + GEL-PVA-ESA	3.55 ± 0.17 ^c^	3.33 ± 0.16 ^d^	10.43	1.894 ± 0.090 ^c^	0.152 ± 0.003 ^a^	0.50 ± 0.02 ^e^
	CPRO + GEL-PVA-OSA	3.21 ± 0.14 ^b^	3.37 ± 0.19 ^d^	9.79	1.778 ± 0.081 ^b,c^	0.197 ± 0.011 ^b^	0.44 ± 0.02 ^c,d^
	CPRO + GEL-PVA-CSA	3.71 ± 0.16 ^c^	4.13 ± 0.20 ^e^	11.55	1.658 ± 0.087 ^a,b^	0.205 ± 0.007 ^b^	0.43 ± 0.01 ^c^

* *n* = 3; SD—Standard Deviation; Different letters (a–h) within the same column indicate significant differences between oxidation parameters of the stored oil samples (one-way ANOVA and Duncan test, *p* < 0.05).

## Data Availability

Data is contained within the article.
